# Renegotiating formal and informal care while ageing abroad: Older Pakistani women's healthcare access, preferences and expectations in Norway

**DOI:** 10.1016/j.jmh.2020.100002

**Published:** 2020-09-29

**Authors:** Sanjana Arora, Melanie Straiton, Astrid Bergland, Bernd Rechel, Jonas Debesay

**Affiliations:** a Faculty of Health Sciences, Department of Physiotherapy, Oslo Metropolitan University, P.O. Box 4 St. Olavs plass, N-0130 Oslo, Norway; b Division of Mental and Physical Health, Norwegian Institute of Public Health, P.O. Box 222, Skøyen, 0213 Oslo, Norway; c European Observatory on Health Systems and Policies, London School of Hygiene and Tropical Medicine, 15-17 Tavistock Place, London WC1H 9SH, United Kingdom; d Faculty of Health Sciences, Department of Nursing and Health Promotion, Oslo Metropolitan University, P.O. Box 4 St. Olavs plass, N-0130 Oslo, Norway

**Keywords:** Older immigrant, Formal care, Informal care, Norway, Healthcare, Access

## Abstract

This study explores older Pakistani women's preferences and expectations of formal and informal care while ageing in Norway. Our study is based on qualitative interviews with older Pakistani women living in Oslo municipality, Norway. The participants were aged between 48 and 81 years and had been living in Norway for 26 to 46 years. Our analyses resulted in five themes: 1) renegotiating the expectations of informal care in light of the ‘nazaam’ (or social system and practices) of Norway, 2) fear of being left behind in residential care homes, 3) disloyalty and shame of being cared for by outsiders, 4) perceptions about the quality of formal care and 5) concerns about mixing with other cultures and genders. Our findings point to potential barriers beyond culture that influence older Pakistani women's preferences for, expectations of and access to formal care services. The study further highlights the structural barriers that older Pakistani women perceive and experience in accessing formal care services in a majority society.

## Highlights

• Older immigrants’ needs and preferences for care are insufficiently known.
• We explore the situation in Norway, a country with a generous welfare state.
• Traditional ideas of care clashed with the availability of formal care.
• Symbolic boundaries were constructed between Pakistani and Norwegian care practices.
• Overall, there is a need to increase knowledge about formal care services.

## 1 Introduction

Although the proportion of older immigrants in Europe is relatively low today, it is expected to rise markedly in the future (Ruspini, 2009; Ciobanu et al., 2017). Thus, care for older immigrants is an important issue, particularly for older immigrant women, since studies have shown that the intersections of gender, old age, ethnicity and socioeconomic factors have implications for health and access to care (Northwood et al., 2018; Småland Goth and Berg, 2011; Villatoro et al., 2018; Brenner and Clarke, 2018). However, despite these trends, we have limited knowledge about immigrants’ needs and preferences for care as they age.

The demographic trend of an increasing number of older immigrants can be witnessed in many countries. In Norway, only five per cent of immigrants were 70 years or older in 2018, but this proportion is predicted to increase to 25 per cent by 2060 (Statistics Norway, 2018). Norway is recognised for its generous social welfare system, in which care for older people is primarily a municipal responsibility and includes both home-based care and nursing homes. The focus of the care policy in Norway, as in other European countries, is that older people should reside at home as long as possible (Helse og Omsorgsdepartementet, 2008; Utredninger, 2005; Christensen, 2003). Previous studies have shown that, overall, immigrants appear to underuse publicly financed long-term care (Bolzman et al., 2004; Hansen, 2014). For example, relatively few older members from ethnic minority groups in Nordic countries live in long-term care facilities, such as nursing homes (Plejert et al., 2014); instead, they are likely to rely on their family for informal care (Hansen, 2014; Schans, 2008). Studies suggest that older immigrants often prefer support from their children (Hansen, 2014; Schans, 2008) over formal support. Moreover, there is a common view that immigrants prefer caring personally for their older family members or that they derive more satisfaction from caring for their older family members than natives (Rapp and Chao, 2000; White et al., 2000; Roff et al., 2004). Filial responsibility is often assumed to be the norm among ethnic minority populations (Gelfand, 2003). Thus, in terms of social relations and caregiving, older adults from ethnic minority backgrounds are not always associated with the disadvantages of being a burden of care (Torres, 2019). However, even if family-based care is more common and perceived as more desirable in immigrant populations, changes in the family structure, responsibilities and roles may make this difficult or sometimes impossible (Curtin et al., 2017), and caregiving by family as a norm is thus increasingly becoming difficult to uphold. This is also one of the reasons why the demand for formal care services for older immigrants is expected to increase in the future (Ingebretsen, 2011; Nergård, 2009). Given this context, the ability of the Norwegian system to adapt to older immigrants’ needs is important for this population to effectively utilise care.

It should be noted that older immigrants are a heterogeneous group with different trajectories, circumstances and care preferences (King et al., 2017). However, the literature on social relations and caregiving does not take into consideration the different social and cultural contexts that influence caregiving among families (Torres, 2019). Moreover, ethnicity and culture are often taken as explanatory variables accounting for the need (or lack thereof) for formal care services for older immigrants. Thus, we know very little about the preferences and expectations of older immigrants regarding their future care in Norway.

## 2 Older Pakistani immigrants in Norway

In this article, we focus on older Pakistani immigrant women, one of the largest groups of older people in Norway (Ingebretsen et al., 2015). The first Pakistani immigrants arrived in the late 1960s and 1970s as labour immigrants. Later, many people, especially women, immigrated through family reunification (Brochmann and Kjeldstadli, 2008). The Pakistani ideals of family care are based on the notions of duty (*farz*) and responsibility (*zimedari*) (Harriss and Shaw, 2010). While acting according to these values may be feasible for some people, the stereotypical assumption that South Asians always look after their own older family members can negatively influence the delivery of welfare services (Atkin and Rollings, 1996) to this population. For example, contrary to the common perceptions about joint living arrangements between children and parents in Pakistani families, a survey on the living conditions among immigrants in Norway found that only 11 per cent of Pakistanis reported living together with their parents, mother or father, 45 per cent reported daily contact, 34 per cent reported weekly contact and the remaining reported monthly or yearly contact (Sandnes, 2016).

Furthermore, within the older Pakistani community, significant gender differences exist between men and women when it comes to language skills, education and employment (Wiggen, 2016; Kumar et al., 2008), in the sense that Pakistani women are more socially disadvantaged than men. In this article, we aim to explore older Pakistani women's preferences and expectations of formal and informal care while ageing in Norway.

## 3 Care options in Norway

Care for older people in Norway is primarily a municipal responsibility. The municipalities operate nursing homes, provide home-based care and determine the type of service and amount of care required for individual users. There are few private providers, and these mostly operate on the basis of contracts with municipalities (Forland, 2009).

In home-based care, the users are divided into three groups on the basis of the type of service they receive: users that receive practical help, users that receive home nursing care and users that receive both practical help and home nursing care. When home care can no longer meet the needs of users, residential nursing homes may become an option, and residents in nursing homes receive around-the-clock services (Borge and Haraldsvik, 2009). Home-based care and institutional care for older or disabled people require means-tested, high cost-sharing of up to 85 per cent of personal income (Ringard et al., 2014).

Although little register-based information on ethnicity and the use of healthcare services is available in Norway (Debesay et al., 2019) a report from 2008 showed that only 2.5 per cent of older immigrants received practical assistance, and three per cent received home nursing care. Those with the longest stay in Norway used home care services more frequently (Nergård, 2008). The care service most commonly requested by people of ethnic minority backgrounds is home nursing care (Ingebretsen, 2011).

Overall, immigrants have been found to make more use of home services than they do of institutional care, but even in the home service sector, they constitute a small minority (Nergård, 2008). The lower use of formal care services by immigrants (Bolzman et al., 2004; Hansen, 2014; Debesay et al., 2019) however means that immigrants are likely to rely on their family for informal caregiving (Hansen, 2014; Schans, 2008). This further underlines the importance of understanding immigrants’ preferences and expectations of informal and formal care while growing old in Norway.

## 4 Methods

This article is based on qualitative interviews with older Pakistani women living in Oslo municipality, Norway. We chose Oslo as our study site, as the highest number of older Pakistanis in Norway reside there. The participants were recruited through snowball sampling, including meeting women at a local mosque and an activity centre, and through key informants. The recruitment criteria were being a Pakistani immigrant woman aged 45 years or older, being permanently settled as a legal resident in the municipality and having lived in Norway for at least ten years. We considered participants aged 45 years and above as older, as age is a social construct and there are no universally defined criteria for who constitutes an old person (Fealy et al., 2012). In total, we conducted 16 interviews and one focus group discussion (FGD) with seven participants.

The participants were aged between 48 and 81 years. Fourteen participants lived in joint living arrangements with either their sons (married/unmarried) or daughters (unmarried), and one lived with her grandson. Nine participants lived in nuclear living arrangements without children, one of whom did not have children of her own. The participants had been living in Norway for 26 to 46 years.

We developed a semi-structured interview guide to explore the participants’: 1) perceptions and experiences of being cared for by their children, 2) expectations of being cared for by their children in the future and changes in care arrangements, 3) coping strategies and 4) perceptions towards residential care homes and home care services. The interviews were conducted by the first author, a PhD student who has experience of conducting research on topics related to healthcare among immigrant women in Norway and is an immigrant woman of South-Asian origin. The interviews were thus conducted in the language preferred by the participants (i.e. Urdu or Punjabi). They were conducted in the participants’ homes, cafes or parks with the first author and participant present. They were recorded and transcribed verbatim. The interviews were then translated into English by both the first author and a professional translator. The data was analysed using Braun and Clarke's (2006) six-phase guide to identify, analyse and report the patterns in qualitative data. The guide entails the following steps: a. familiarising oneself with the data, b. generating initial codes, c. searching for themes, d. reviewing the identified themes, e. defining and naming the themes and f. preparing the report. First, all the transcripts were read and a first set of codes was generated. Then, we combined all the similar codes and quotes while labelling them in clusters and organising them into themes. Upon discussion, we reviewed the themes again and merged them into smaller themes.

The study was approved by the official body Data Protection at the Norwegian Centre for Research Data (project number: 52,078). All participants received written and verbal information about the study and provided informed consent. The participants were informed about the possibility to withdraw their consent at any point of the study, without any consequences. All the participants’ names used below are pseudonyms.

We use the term residential care homes to refer to nursing homes, old age homes or communal living spaces for older people in need of care. Professional home care services include both home nursing care and practical assistance.

## 5 Findings

Family, mainly children, was reported by all participants as the main preference for care in old age, and caregiving by children was viewed as an extension of living together. Most participants who were residing with children felt that living together was integral to their physical, social and emotional support.

The analyses resulted in five themes: 1) renegotiating expectations of informal care in light of the nazaam (or social system and practices) of Norway, 2) fear of being left behind in residential care homes, 3) disloyalty and shame of being cared for by outsiders, 4) perceptions about the quality of formal care and 5) concerns about mixing with other cultures and genders.

### 5.1 Renegotiating expectations of informal care in light of the nazaam of Norway

Living separately from children was associated with the uncertainty of receiving informal care. Thus, all the participants based their expectations of informal care on their current living circumstances.

The participants reported trying to renegotiate their expectations of receiving informal care in the future. This was due to the nazaam (or social system and practices) of Norway. The differences in lifestyle, preferences for the nuclear family, differing roles of women (or primary caregivers), their children's own childcare responsibilities and the social welfare system in Norway enabled the children to live separately from their parents and to be less involved in informal care than in Pakistan.

Yasmin, who, with her husband, lived with her son and daughter-in-law, reported her frustration and stated, ‘There is the nazaam here (in Norway), like we have our nazaam there (in Pakistan) that we are going to live together. But no, here the nazaam is of staying in their own homes, to get their own houses and live there. Here this is exactly the dark point of here (Norway)!’

Furthermore, a few participants attributed the sense of independence among children in Norway as the reason for the uncertainty of their living together and for the enabling of care from outsiders, as pointed out by Ameena, who lived with her son and daughter-in-law, along with her husband: ‘They (older people) get lonely, they get left out alone, this is why they (older people) go there (care home), they get sent there. From the beginning, the children are taught independence…It's fine until they grow up, then they think, we are independent, we can do this (live away from parents).’

While some expressed their lack of control over the nazaam in Norway, they also reported having come to terms with it, despite perceiving it to be in contradiction with their culture. For example, Saba, whose son and daughter-in-law lived nearby but in a separate household, reported: ‘Earlier, my nature was that of an angry person. Now the anger has gone. You can say that it is a kind of compromise. Okay? Of course it hurts when the child, it is in our culture that when children leave, it hurts, there is loneliness.’

Saba further spoke about her own adjustment of expectations, while simultaneously pointing out the inability of other women, particularly those who had sons in the community, to cope with such change: ‘Here there are many others who would say “Oh, my son has left me!” They would cry and whine! Some say I should have been childless…Some say my son left me, I made him a doctor, made him an engineer.’

Since the primary responsibility of daily caregiving was expected to be conducted by daughters-in-law, the participants with sons expressed a greater struggle in renegotiating their expectations and perceptions about informal care than the participants who had only daughters; the latter seemed better prepared for nuclear living arrangements and the use of formal care services.

Zara, whose son and daughter-in-law both had full-time jobs and lived separately, reported that the burden of caregiving usually fell on women, particularly daughter-in laws who are expected to move in with the husband and his family in Pakistani families. This was thought to be a common source of conflict in family relationships. The different roles of women in Norway, due to their greater involvement in the labour market as compared to Pakistan, were also perceived to be a result of the different nazaam in Norway. Zara accepted living separately from her son and daughter-in-law, understanding that joint living arrangements and subsequent informal care was not as compatible with the nazaam of Norway as it was in Pakistan. The gender of the children thus influenced older Pakistani women's perceptions towards formal care services.

Despite the negative conception of the nazaam in Norway vis-a-vis Pakistan, some participants also reflected on the positive implications of a social welfare system which allowed their children to be less involved in informal care but also ensured a safety net of formal care in old age through formal care services. They compared the norm of joint family living and informal care in Pakistan with the benefits of equal and universal access to formal care for older adults in Norway. For example, Hina, whose unmarried daughters lived with her and her husband, stated (in Pakistan), ‘it's like if children go away, then parents have nothing and then they are also gone! (miserable). If they have money, then it's fine but, if not, then they are just gone! Here they give (help) to everyone.’

Furthermore, in the FGD, we found that all the participants believed that, as women, they had accepted their new living and daily care arrangements better than the men in their community. They attributed this to Pakistani women's openness to talk about such topics amongst themselves and their concern about burdening their children, as compared to Pakistani men, who, they believed, still held on to the notions that the women had left behind (in Pakistan).

Faced with the reality of a different nazaam in Norway, the participants reported different perceptions towards residential care homes and professional home care services. Although both were perceived to be less preferable than informal care, overall, the participants perceived professional home care services to be more acceptable than residential care homes for older people. However, their perceptions of both varied considerably. The themes below highlight their perceptions towards both types of formal care services: residential care homes and professional home care services.

### 5.2 Fear of being left behind in residential care homes

Most participants, whether living jointly or separately from their children, reported concerns of being left behind in a residential care home in the future. Selma, who lived with her husband and unmarried son, noted ‘what do we think [about institutional care]? The heart hurts thinking that this person got left behind. We fear that our turn will come! It hurts.’

Some participants reflected on the possibility that, with declining health, residential care homes might become a necessity, as Saba pointed out: ‘We ourselves hope that such a time should never come, that we have to go there (a care home). But when the conditions are not favourable there (in Pakistan), or here (in Norway), then it's a necessity, then what can be done!’

Another participant, Bushra, who lived with her unmarried daughters and her husband, also spoke about the possibility of a residential care home as a last resort. She had plans to prepare her home for her older age and declining health: ‘If I am bedridden, then the children cannot take care of me. Then of course I will have to go there. But I am getting my home constructed and installing an elevator on which I can sit and go up.’

Some participants were also concerned about feeling lonely in care homes, as Sarina reflected: ‘I sometimes think that if I go to a care home, then my children won't even have time to even visit me. They may come after several weeks.’ We found that all the participants associated residential care homes with loneliness and abandonment in old age.

### 5.3 Disloyalty and shame of being cared for by outsiders

In general, the participants associated the idea of loyalty of children towards their parents with informal care, which symbolised, for both Pakistani children and parents, a good upbringing worthy of respect from the community. Some participants conceptualised living together as a mark of their children's loyalty, in contrast to not being cared for by their children, which was associated with disloyalty and shame, reflecting abandonment by the children. For example, Fatima, who lived in a large household with three sons and one unmarried daughter, expressed that she had never thought about the possibility of her children not caring for her and believed that ‘those who have good children would not send their parents to care homes.’

Some participants also perceived that Pakistani children are more caring towards their parents than Norwegians, as Selma noted: ‘If children are loyal, then they can't send them there (care home), right? If children are sensible, then they will take care of parents. No other option for poor Norwegians…their children don't care much; the Pakistanis don't have children like that.’

Moreover, these ideas of disloyalty on the part of children were associated with stigma and subsequent shaming by community members. Selma further spoke about the negative remarks children might hear from the Pakistani community in Norway, as well as in Pakistan, if they did not care for their parents: ‘If one gets old and children don't take care of their parents, then our culture is such that they would be scolded by others…when they go to Pakistan or anywhere, they will say you didn't care for your parents and are showing your face here now?’

However, some participants who did not live with children felt that the perception of the Pakistani community towards formal care, specifically residential care homes, might change in the future. For example, one participant, Ameena, believed that the next generation of children are ‘similar to Norwegians,’ have ‘better’ values and would not gossip about someone going to a care home.

However, none of the participants associated the use of professional home care services with feeling abandoned, and this type of service was perceived in a more positive light than residential care homes. Some women, though, reported having insufficient information about how to access such services or not knowing anyone who received home care services. They further believed that the use of professional home care services within families was often kept confidential.

### 5.4 Perceptions about the quality of formal care

Concerns about the quality of formal care were brought up in the context of both residential care homes and professional home care services. The participants perceived that care homes were for the severely ill and immobile and therefore did not think they were suitable for them in the immediate future. Nonetheless, some participants were concerned about the quality of care available at the care homes. For example, Saba, who reported that members of the Pakistani community had now started to live in care homes, believed that care to be loveless: ‘The love, or I mean, the way children have care for their loved ones, they (professional carers) cannot have that, right? They just have to do their duty…this is natural.’ Another participant, Selma, who also believed the quality in care homes to be poor, noted*:* ‘I have an acquaintance who works there and she tells me that they do not care in a good way…The family members come to visit rarely, and those workers can only give them medicines and make them sleep. But the people want someone to talk to them, for the family to come, poor them! They keep hoping that someone will come.’

Contrary to this, a few participants believed that residential care homes might actually be better equipped to care for older people, since professionals would be doing the care duties instead of their children, with their busy lives, jobs and responsibilities of caring for their own children. This was specifically the case for the participants who had daughters, such as Bushra, who noted, *‘*Like I have done my duties at work today, the same way those people (at care homes) are also going to do their duties. Though I have never been there, and I have seen old Norwegians, so it seems nice, but I have never been there myself. And I have also not visited anyone from our country there.’

Concerns over the quality of care in professional home care services were also raised by a few participants. They felt that the home care professionals did not have enough time during their visits, and they reported feeling that they did most of the work themselves that they had expected to get support for from the professionals. One participant even reported hiring a private home care professional in lieu of a public one, as she believed the former took care of her as a family member would: ‘She was from Nepal; when I called her for the first time, obviously I had to give her money, but then she hid inside a room…she used to say “I am taking care of you the way I would have taken care of my own mother.”’

Even when some participants accessed professional home care services, they were left dissatisfied, as they struggled to achieve continuity in such care. For example, one participant described how she felt frustrated when an official from the municipality, who visited her home, insisted she should try putting on her compression socks on her own. The participant reported exasperation as she was unable to put on the compression socks by herself, yet they continued to insist that she tried. Thus, personal ‘bad’ experiences of encounters with professional home care, although there might be good reasons for the potential disagreements between the parties, undoubtedly shape older women's perceptions of the quality of professional home care.

### 5.5 Concerns about mixing with other cultures and genders

Some participants perceived that care from outsiders, both in the form of residential care homes and professional home care services, was difficult to relate to culturally. For example, when asked about her perception of residential care homes, Yasmin, pointing to a building from her window, said, *‘*So this belongs to the European people, I mean the ones who are alone, then they have to come here to stay.’ This statement highlights the alienness of the concept of residential care homes for the participant and their association with European people.

This cultural difficulty in relating to residential care homes was further heightened by worries about being able to continue their daily routines, speak their own language and eat their own foods, irrespective of their Norwegian language skills or extent of socialising with Norwegians. As a result, most participants voiced their desire to have a separate care home or a separation within the building for Pakistani, or Muslim, older people. Below is an excerpt from the FGD, highlighting the view that there should be a separation for women or for Muslims.

We have to pray, we don't want to watch the TV which the Norwegians watch. So there should be a separation. We won't say that, okay, make a different care home for Pakistanis altogether and that only Pakistanis should live in that. No, I would never want that. So it's like there should be a care home which has a separation, such that, okay, this is the partition for women, the women are roaming around here, this is the bedroom, this is the sitting room, this is their prayer room…and it is obvious that they eat halal food…they should have their own TV. I said if you give us own TV, give us also Pakistani channels, and then our time would pass very nicely.

Most participants in the FGD agreed with this view, except one who stated that she would not have any issues living in the same care home as the Norwegians, as she already socialises with them in daily life.

With regard to professional home care services, a few participants, despite speaking Norwegian well, were concerned about receiving care from Norwegian home care professionals due to different customs or norms: ‘There can be a clash of culture. Maybe when I really need it, I would think carefully about it and prepare my mind accordingly, but for our people, for example, it's obvious that we Muslims don't shake hands. I mean with men; but in this culture, we shake hands with Norwegians, but not with our own.’ Moreover, most participants reported comfort in the possibility of finding ‘their own people’ as home care professionals, which they believed could make them feel more comfortable in their homes.

Gender was also significant in influencing their perceptions about both care homes and professional home care services. The participants expressed their concern about living in a care home as a woman, as a participant in the FGD pointed out: ‘It is better for us that there should be one (care home) for women. See, all we women would be there, we will be older. We don't know if we would be able to dress on our own or not, don't know in which state we would be roaming around.’ Thus, in the FGD, some participants expressed that they would feel more comfortable in a gender-divided care home or a care home with a separation for either older women or Muslim women, while others seemed surprised to learn that men and women dwell in the same care home.

Regarding professional home care services, while most women felt that having a male home care professional would be uncomfortable for them, they coped knowing that other family members would be present in their homes and they would not be alone. The gender of the professional became relevant mainly when the care required physical touch or any form of intimate care, as highlighted by the FGD participants:

P1: My aunt was ill. So the men (home care professionals) would come and change her pads…they would clean her up. We did not like it. Because we are Muslims. But it was their job.

P2: A little bit is okay when a person gets old, so then if a man changes or a woman changes (the pads), but no not me. Not for me!

P3: Not for our women!

Thus, consideration of their being elderly did not entirely decrease their discomfort with being cared for by a male home care professional. Another participant who lived alone reported feeling uncomfortable with the presence of a Pakistani male professional in her home, who generally assisted with daily housekeeping and cooking. As a result, she decided to discontinue receiving care from him and was allocated a Norwegian female home care professional. Thus, both gender and cultural concerns seemed to be relevant for the participants’ future choice of care, depending on the nature of the care and the participants’ preferences.

## 6 Discussion

The aim of this article was to explore older Pakistani women's expectations of and preferences for future care. In our exploration, we shed light on the underlying tension between their traditional notions of informal care and formal health care arrangements.

Our study revealed several factors which influenced participants’ access to formal care services, the most significant of which related to their ability to seek formal care services. The ability to seek care encompasses cultural and social factors that influence the possibilities to access services and corresponds to the dimension of the acceptability of services. It also includes perceptions about the quality of services, personal autonomy and the capacity to choose to seek care, as well as knowledge about health care options and individual rights (Levesque et al., 2013).

Firstly, we found that participants associated informal care with loyalty and respect in the Pakistani community, which highlights the role of culture in influencing the norms of caregiving and the ability of participants to seek formal care services. This is consistent with previous research in Norway, which found that the willingness or ability of the son to care for the older family member was the basis for respect and honour among the Pakistani-Norwegian men's community (Næss and Vabø, 2014).While herein culture could be construed as influencing the ability to seek formal care services, it also highlights how the minority caregiving practices are organised and constructed in society by the minority members themselves. What is striking about the construction of such a normative ideal of caregiving is the symbolic boundary that the participants create between Pakistani and Norwegian practices (‘our’ vs. ‘theirs’). This finding is consistent with how British Pakistanis have been found to conceptualise family care as a morally-laden practice to define themselves in contrast to ‘English’ society (Harriss and Shaw, 2010). In Norway too, familial care has been found to be regarded as a highly morally-imbued practice which works to define Norwegian-Pakistanis as a community that differs from Norwegian society (Næss and Vabø, 2014). It seemed that by invoking their normative ideals of care, they attempted to de-stigmatise themselves by viewing their own form of caregiving to be morally superior to that being practised in the Norwegian society. Thus, while normative ideals of caregiving based on cultural practices influenced the acceptability of formal care services, they also functioned as a strategy to renegotiate their own identity positively within the majority society.

It should be noted, however, that, even though the participants perceived familial caregiving as a normative ideal, the preference for this type of care has also been found amongst older adults from majority populations (de Graaff and Francke, 2003; Suurmond et al., 2016; Grady, 2014). Thus, it does not seem to be a specific cultural need. In the case of our participants, the preference for informal care intersected with the lack of familiarity, as well as the struggle for validation of their family patterns (Harriss and Shaw, 2010), as discussed below.

The lack of familiarity existed due to the participants experiencing a transition to their new circumstances in Norway as older members of an ethnic minority. Firstly, it led to the fear of loneliness and of being cared for by outsiders, specifically in the case of residential care homes. Participants found comfort in the possibility of finding Pakistani home care professionals if needed versus being in the largely unfamiliar setting of a care home. Our study found that participants’ fears of being lonely and abandoned in care homes influenced their acceptance of such services. Feelings of loneliness have been found among older adults living in professional care homes in general (Drageset et al., 2012). Studies have shown that older Norwegians, in general, experience loneliness and loss of agency after moving into professional care homes, due to a combination of health problems, poor care and loss of family and friends (Iden et al., 2015). Further, loneliness, lack of social support and the perceived inadequacy of care among older people residing in nursing homes have been found to be associated with depression (Jongenelis et al., 2004) and mortality (Drageset et al., 2013). Thus, the acceptability of care homes is a concern among older adults in general. However, for the Pakistani women in our study, this fear of loneliness was heightened, possibly due to the lack of cultural comforts, such as food, language or TV channels, that could help them maintain a minimal sense of familiarity. In general, older adults who live in care homes desire to maintain a sense of home through familiar building and interior design, as well as eating and drinking practices (van Hoof et al., 2016; Rijnaard et al., 2016). Understandably, being a minority seems to influence the experience or anticipation of loneliness for some older Pakistani women. This, in turn, might influence their ability to access such services.

Secondly, the participants also experienced unfamiliarity as an ethnic minority due to how they perceived the nazaam of Norway, i.e. the social system and practices. The nazaam was interpreted as an enabler for formal care, contradicting how the norms of joint living arrangements, the role of daughters-in-law as primary caregivers and the subsequent caregiving would be typically organised in Pakistan. This highlights how previously held perceptions of living arrangements and informal care lead to the struggle that some older Pakistani women experience when trying to renegotiate their expectations of informal care while ageing in Norway. This tension is hardly due to rigid cultural conceptions of informal care, but most likely due to the struggle for validation of family patterns of living and caregiving in the majority society. Because of the challenge to find validation in a society and a welfare regime based on majority practices, older immigrants might be institutionally disadvantaged (Harriss and Shaw, 2010). Thus, the lack of familiarity, the fear of loneliness, perceptions about the nazaam of Norway and the struggle for validation for their living and caregiving practices point to potential barriers in older immigrants’ ability to seek residential care home services.

However, we found that cultural factors did not solely affect access to professional home care services, as the participants pointed out the possibility to find ‘their own people’ who worked as home care professionals. However, some participants seemed to suggest a potential culture clash with male Norwegian home care professionals. Indeed, cultural concerns became especially significant when participants considered the gender of the home care professional. Thus, concerns about both gender and culture influenced their ability to engage in the use of home care services, as exemplified by one participant who did not want to continue with the male Pakistani home care professional and instead requested care from a female Norwegian home care professional. Moreover, when it came to intimate care, consideration of their older age did not seem to decrease the discomfort they felt in being cared for by a male home care professional. Thus, for both professional home care services and care homes, gender influenced the acceptability of formal care services. Participants perceived that being a Pakistani Muslim woman affected their level of comfort with male care professionals either in their own home or in a care home setting.

It is widely known that concerns about the gender of care professionals exist among care home residents (Hajek et al., 2017) in general, but, in the Pakistani case, cultural considerations also seem to have an impact on their acceptability of receiving formal care services from men.

Interestingly, the women believed that Pakistani men would be more resistant towards new nuclear living arrangements and subsequent informal care. The women perceived themselves to be more understanding around the burden informal care would put on their children. This is in contrast with a study in Norway which showed that Pakistani men believed that first-generation women had more difficulty accepting the idea of a care home, since they had remained confined to their household and spoke less Norwegian (Næss and Vabø, 2014). This gender distinction in perceptions of acceptability of formal care services could be investigated in future research.

As stated earlier, care homes, in contrast to professional home care services, carried the fear of loneliness and abandonment. Participants believed that ‘being a Pakistani’, their culture facilitated construction of a normative ideal of caregiving, the failure of which invoked shame from the community. At the same time, the perception that the second generation/their children will become ‘similar to Norwegians’ was believed to reduce the stigma associated with care homes and thereby improve their approachability. Thus, through drawing a symbolic boundary of Pakistani practices of familial caregiving vis-a-vis Norwegian ones, some participants conceptualised their caregiving practices as superior to those of the Norwegians, but for others, the failure to fulfil the normative ideal of caregiving also invited stigma from the Pakistani community. Moreover, none of the participants associated professional home care services with abandonment and loneliness, possibly since the use of such services facilitated living in their own homes. It is possible that the participants perceived living together with their children as central to their normative ideals of familial caregiving, irrespective of whether daily caregiving was managed by the children or professionals. However, barriers beyond gender and cultural considerations still persisted in access to professional home care services. Some participants reported the lack of information as a barrier to access, as they believed such information was kept private within families. Previous studies have found that the use of such services is often not discussed in public by Norwegian Pakistani men because it might convey the unwillingness of their children in providing caregiving (Næss and Vabø, 2014). It is possible that this lack of information hindered the approachability of professional home care services (Levesque et al., 2013). Approachability relates to the fact that people with health care needs can actually identify that some form of service exists and that they can reach it through the availability of information, and it thus relates to people's ability to perceive a need for healthcare (Levesque et al., 2013). We found that the difficulty participants experienced in perceiving the need for care due to the lack of information, as well as in seeking care due to gender and cultural considerations, in general hampered their access to services.

Concerns about the quality of care were found to exist for both care homes and professional home care services. Regarding care homes, concerns around the quality of care were associated with the idea of carers doing their professional duty. For some, perceiving carers as professionals doing their duty implied care devoid of love, and they thus held a negative perception of such care. Contrastingly, for a few participants, this implied that the professional carers would do their jobs effectively, which they perceived as positive. Previous studies in Norway have found that Norwegian-Pakistanis dread the thought of public care (especially care homes) because they fear inadequate support and familial detachment, although many acknowledge that such care might be necessary in light of familial or pragmatic circumstances (Næss and Vabø, 2014). Similarly, it has been found that although family caregivers of older Pakistani immigrant women generally saw providing care for their older relatives to be a duty, they also shared the view that such expectations were hard to reconcile with their life in Norway (ref: current authors). In our study, we found that this was specifically the case for participants such as Bushra who had daughters and had positive perceptions towards the quality of care in care homes. Moreover, participants with daughters, such as Hina, felt that the positive implications of the social welfare system and the resulting formal care services outweighed the negative implications of the nazaam of Norway. This could possibly be due to the tradition of daughters-in-law being the main caregivers in South-Asian families (Ahmad, 2012; Gupta and Pillai, 2012). The participants who only had daughters possibly had a higher ability to perceive the need for formal care services, as well as a higher ability to seek those services in the future if needed. Regarding concerns about the quality of care in professional home care services, the lack of sufficient time by professionals, the struggle to arrange the continuity of home care services and the desire for familial forms of care instead of professional ones influenced the participants’ ability to engage with professional home care services, thus leaving them dissatisfied. This relates to people's ability to engage with the healthcare system and includes the participation and involvement of people in decision-making and treatment decisions (Levesque et al., 2013). In this regard, it would be relevant to explore older immigrants’ ideas of rehabilitation and trust towards formal care services in future research.

The participants did not mention any difficulties in their ability to pay for such services, even though a fee is required to use these services in Norway, as the services are means-tested and may require high cost-sharing of up to 85 per cent of personal income (Ringard et al., 2014). It is possible that a general lack of knowledge and information about formal care services influenced their perceptions of the affordability of such services. Similarly, discussion about the availability of professional care services did not come up in the interviews, possibly due to very few participants having been users of professional home care services and none of them using care homes.

The strength of the article lies in providing evidence on preferences and expectations of older Pakistani women, a demographically significant group, regarding their future care in Norway. While this study offers rich data in the form of participants’ in-depth experiences, it is important to take into account the heterogeneity of immigrant groups, as well as their specific circumstances based on age, gender, and culture, among others, in order to infer transferability to other immigrant groups in the Norwegian healthcare context. Our findings might not apply for all Pakistanis, for instance, in our sample 14 out of 23 participants reporting living with their children. This is in contrast to the living conditions survey among immigrants in Norway, which found that 11% of Pakistani immigrants reported living with parents (Sandnes, 2016).

Another limitation of this study relates to the interviewer's insider position. While having an insider position can have its methodological advantages, it also presented challenges of assumptions of shared understanding (Kanuha, 2000). To minimize this, frequent discussions with co-authors from different thematic backgrounds, locally and internationally, as well as writing a reflexive journal helped us keep check of the sources and understanding behind certain interpretations.

## 7 Conclusion

This study points to the potential barriers beyond culture that influence older Pakistani women's preferences for, expectations of and access to formal care services. It further highlights the structural barriers that older Pakistani women perceive and experience as an ethnic minority in accessing formal care services even in a rich European country like Norway with a generous welfare state. Despite the availability of formal care services, access to these services is not necessarily possible for all, and the barriers could be invisible under the dominant narrative of the cultural patterns of informal care. It is therefore important to improve the familiarity of formal care services for ethnic minorities by making these systems more compatible with their needs and preferences. Lack of information hinders the approachability of formal care services, a situation that is likely to exist in other European welfare states with well-established formal care sector and an increasing share of older immigrants. Thus, in order to improve the ability of older ethnic minority members perceive a need for, and to seek and engage with formal care services, interventions should focus on improving knowledge about such services.

## Statement of ethical approval

This study was approved by the official data protection body at the Norwegian Centre for Research Data (Project number: 52078)

## Statement of funding

This study is part of a PhD project at Oslo Metropolitan University

## CRediT authorship contribution statement

**Sanjana Arora:** Conceptualization, Formal analysis, Writing - original draft, Writing - review & editing. **Melanie Straiton:** Writing - review & editing. **Astrid Bergland:** Writing - review & editing. **Bernd Rechel:** Writing - review & editing. **Jonas Debesay:** Conceptualization, Formal analysis, Writing - original draft, Writing - review & editing.

## Declaration of Competing Interest

The authors declare that they have no competing interests.
